# Sequence analyses of fimbriae subunit FimA proteins on *Actinomyces naeslundii *genospecies 1 and 2 and *Actinomyces odontolyticus *with variant carbohydrate binding specificities

**DOI:** 10.1186/1471-2180-6-43

**Published:** 2006-05-10

**Authors:** Mirva Drobni, Kristina Hallberg, Ulla Öhman, Anna Birve, Karina Persson, Ingegerd Johansson, Nicklas Strömberg

**Affiliations:** 1 Department of Odontology/Cariology, Umeå University, SE-901 87 Umeå, Sweden

## Abstract

**Background:**

*Actinomyces naeslundii *genospecies 1 and 2 express type-2 fimbriae (FimA subunit polymers) with variant Galβ binding specificities and *Actinomyces odontolyticus *a sialic acid specificity to colonize different oral surfaces. However, the fimbrial nature of the sialic acid binding property and sequence information about FimA proteins from multiple strains are lacking.

**Results:**

Here we have sequenced *fimA *genes from strains of *A.naeslundii *genospecies 1 (n = 4) and genospecies 2 (n = 4), both of which harboured variant Galβ-dependent hemagglutination (HA) types, and from *A.odontolyticus *PK984 with a sialic acid-dependent HA pattern. Three unique subtypes of FimA proteins with 63.8–66.4% sequence identity were present in strains of *A. naeslundii *genospecies 1 and 2 and *A. odontolyticus*. The generally high FimA sequence identity (>97.2%) within a genospecies revealed species specific sequences or segments that coincided with binding specificity. All three FimA protein variants contained a signal peptide, pilin motif, E box, proline-rich segment and an LPXTG sorting motif among other conserved segments for secretion, assembly and sorting of fimbrial proteins. The highly conserved pilin, E box and LPXTG motifs are present in fimbriae proteins from other Gram-positive bacteria. Moreover, only strains of genospecies 1 were agglutinated with type-2 fimbriae antisera derived from *A. naeslundii *genospecies 1 strain 12104, emphasizing that the overall folding of FimA may generate different functionalities. Western blot analyses with FimA antisera revealed monomers and oligomers of FimA in whole cell protein extracts and a purified recombinant FimA preparation, indicating a sortase-independent oligomerization of FimA.

**Conclusion:**

The genus *Actinomyces *involves a diversity of unique FimA proteins with conserved pilin, E box and LPXTG motifs, depending on subspecies and associated binding specificity. In addition, a sortase independent oligomerization of FimA subunit proteins in solution was indicated.

## Background

*Streptococcus *and *Actinomyces *species *, e.g. A. naeslundii *genospecies 1 and 2 and *A. odontolyticus *(referred to as species), constitute a large portion of the commensal microflora on oral surfaces [1,2]. While *A. odontolyticus *dominates at tongue surfaces, *A. naeslundii *genospecies 1 and 2 colonize plaque and buccal surfaces but with different patterns [1,3,4]. Moreover, *Actimomyces *species have been implicated in dental caries, periodontitis and other infections [5-8].

Besides adhesion to salivary pellicles and oral epithelial surfaces [9,10], actinomycetes and streptococci participate in inter- and intra generic coaggregations, as defined by the *Actinomyces*-*Streptococcus *coaggregation groups A to F for *A. naeslundii *genospecies 1 (*i.e. *groups B, C and D), genospecies 2 (*i.e. *groups A and F) and *A. odontolyticus *(*i.e. *group E)[9,11]. To participate in these adhesive interactions, *Actinomyces naeslundii *genospecies 1 and 2 express two antigenically different fimbriae, type-1 and type-2 [12-14]. Type-1 fimbriae bind to acidic proline-rich proteins and statherin in salivary pellicles on teeth [13,15]. Type-2 fimbriae contribute to adhesion and colonisation [13] by binding to Galβ structures (*i.e. *β-linked galactose or galactosamine) [16] in salivary pellicles [17], streptococci [18], oral epithelial cells [19,20] and to polymorphonuclear leucocytes [21]. Both genospecies 1 and 2 express type-2 fimbriae but with variant Galβ binding specificities [14,20], and each genospecies exhibits at least two types of Galβ-based hemagglutination patterns [1].

The major subunit genes of type-2 and type-1 fimbriae, *fimA *and *fimP*, respectively, have been cloned and sequenced from *A. naeslundii *genospecies 1 (strain 12104) and 2 (strain T14V) [22-26]. The deduced FimA and FimP subunit proteins are 534 and 533 amino acid proteins, respectively, with 34 % amino acid identity. FimA and FimP contain seven conserved proline-containing regions involved in folding of the two proteins and an LPXTG sorting signal followed by a N-terminal membrane spanning domain [25]. Structurally diverse *fimA *and *fimP *genes, as well as species-specific *fimA *gene segments, have been found for *A. naeslundii *genospecies 1 and 2, and linked to different coaggregation groups and types of Galβ- and PRP- related adhesion properties [14,27]. However, the *fimA *gene has so far only been sequenced from a single strain of both genospecies 1 (12104) and 2 (T14V) [24,25].

*A. odontolyticus *is a prominent member on the tongue as well as present at supra- and subgingival sites [1,4]. The fimbrial structure of *A. odontolyticus*, and host receptors, employed for its adhesion have not been fully investigated. However, inhibition studies show that sialylated carbohydrate structures, such as sialyl Tn and 3' sialyllactose structures, serve as a salivary glycoprotein gp-340 receptor for *A. odontolyticus *strain PK984 [28], which is a reference strain for coaggregation group E. Moreover, hybridization studies [14] have indicated FimA- or FimP-related adhesins on *A. odontolyticus*, but *fimA *or *fimP *genes have not yet been identified or sequenced from *A. odontolyticus*.

The aim of this study was to characterize *fimA *genes from several strains of *A. naeslundii *genospecies 1 and 2 with variant Galβ binding specificities and from a strain of *A. odontolyticus *PK984 with a sialic acid binding specificity.

## Results

### A. naeslundii genospecies 1 and 2 and A. odontolyticus PK984 display deviating cell binding properties

*A. naeslundii *genospecies 1 and 2 express type-2 fimbriae with variant types of Galβ binding specificity and *A. odontolyticus *express a sialic acid binding specificity potentially related to type-2 fimbriae (Table 1). Accordingly, while *A. odontolyticus *PK984 agglutinated untreated but not sialic acid-depleted red blood cells, *A. naeslundii *genospecies 1 and 2 (strains 12104 and LY7, respectively) agglutinated only sialic acid depleted cells strongly, due to exposure of penultimate Galβ-residues (Table 1).

### Subtypes of FimA proteins in A. naeslundii genospecies 1 and 2 and A. odontolyticus with different binding specificity

*fimA *genes were amplified by PCR and sequenced from strains representative for genospecies 1 (12104, Pn-22-E, Pn-6-N, P-5-N), genospecies 2 (T14V, P-1-N, P-1-K, LY7) and for *A. odontolyticus *(PK984) with different Galβ and sialic acid binding properties, respectively. In addition, the *A. naeslundii *strains were selected to include two types of Galβ-dependent hemagglutination patterns for each genospecies.

**Figure 1:**
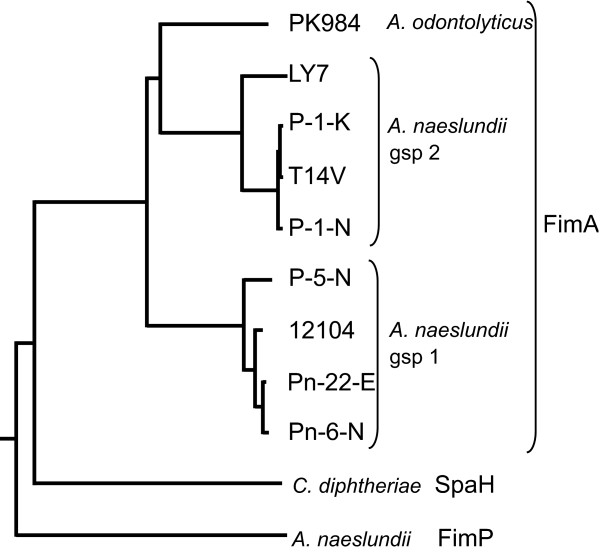
**Dendrogram of FimA proteins**. Sequence analyses were preformed on FimA proteins from *A. naeslundii *genospecies 1 (strains 12104, Pn-22-E, Pn-6-N, P-5-N), *A. naeslundii *genospecies 2 (strains T14V, P-1-N, P-1-K, LY7) and *A. odontolyticus *(strain PK984) using Clustal W. The SpaH protein from *C. diphtheriae *and FimP from *A. naeslundii *LY7 genospecies 2 were included as out-groups.

Structurally variant FimA proteins were found in *A. naeslundii *genospecies 1 and 2 and *A. odontolyticus*, respectively (Table 2, Fig. 1). The FimA sequence identity between the three species was in the 62.8–66.4% range, while the sequence identity between strains of the same species was 88.6–99.6 %. Accordingly, sequence analyses of the FimA proteins, clustered FimA proteins from genospecies 1 and 2 and *A. odontolyticus *into separate groups, but the two latter species more closely (Fig. 1). In addition, the SpaH fimbriae protein from *Corynebacterium diphteriae *clustered more closely to FimA than did FimP from *A. naeslundii*.

### Structural features of the novel FimA protein in A. odontolyticus with a sialic acid specificity

**Figure 2:**
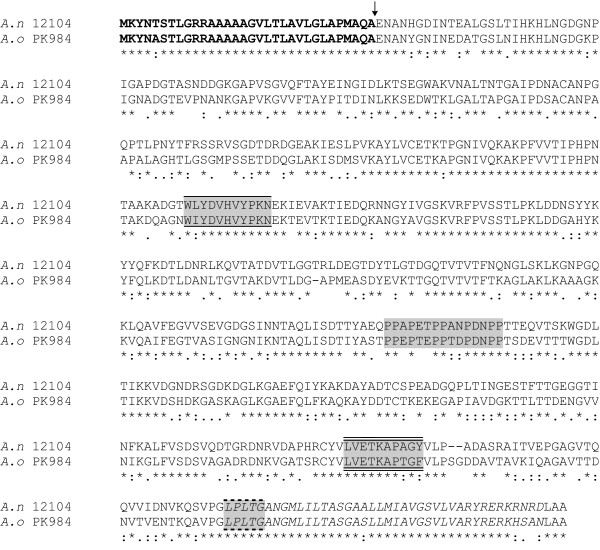
**Sequence of FimA from *A. odontolyticus *strain PK984**. The PK984 FimA sequence was compared to FimA from *A. naeslundii *genospecies 1 strain 12104. Identical (*), conserved (:), and similar (.) amino acids are indicated. Marked are the N-terminal signal peptide (bold typeface) and peptidase cleavage site (arrow), pilin motif (grey box lined with single line), a proline-rich region (grey box), E box (grey box lined with double lines), and an LPXTG motif (grey box lined with dashed line). The motifs/domains are also present in *A. naeslundii *genospecies 2 (not shown).

The *fimA *gene of *A.odontolyticus *PK984 encodes a 535 amino acid protein (Fig. 2). The FimA protein contains: *i*) an N-terminal signal peptide with a signal peptidase cleavage site, *ii*) a pilin motif for polymerisation of subunit monomers, *iii*) a proline-rich segment, *iv*) an E box motif, *v*) an LPXTG sorting motif, and *vi*) a C-terminal cell membrane spanning domain.

Cleavage of the 535 amino acid FimA protein of *A.odontolyticus *between residues 32 and 33 would generate a fimbrial subunit protein of 503 residues. This gives a theoretical subunit molecular weight of 52.6 kDa.

### Species-specific and conserved segments in FimA proteins from A. naeslundii genospecies 1 and 2 and A. odontolyticus

**Figure 3:**
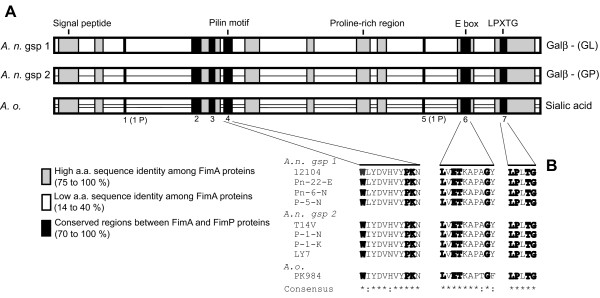
**Comparison of FimA protein sequences from *A. naeslundii*genospecies 1 and 2 and *A. odontolyticus *with different binding properties**. **A) **Shown are conserved regions between FimA and FimP proteins (black boxes) and regions with high (grey boxes) or low (white boxes) sequence identity between FimA proteins. The low FimA amino acid (a.a.) sequence identity regions (unlined, double and triple lined white boxes) are species-specific due to a high strain-to-strain sequence identity for each species. High FimA sequence identity domains include the N-terminal signal peptide, pilin motif, proline-rich region, E box, and LPXTG motif (motifs are marked and the seven conserved proline-containing domains identified by Yeung and Cisar are numbered). The proline-containing domains 1 and 5 of FimA display only a conserved proline residue, in contrast to FimP, in which the entire seven proline-containing domains are conserved. The FimA proteins from genospecies 1 and 2 and *A. odontolyticus *contains 32, 33 and 32 amino acid signal peptides and 534, 535 and 535 amino acid proteins, respectively. **B) **Alignment of the pilin, E-box and LPXTG motifs present in FimA from different strains. Postulated key determinants are in bold [29, 39].

The FimA proteins from strains of *A. naeslundii *genospecies 1 (n = 4) and 2 (n = 4) and *A. odontolyticus *(n = 1) were compared for FimA sequences or segments conserved between the species or unique to each species (Fig. 3A). The generally high sequence identity (>97.2%) within a genospecies (Table 3) revealed species specific sequences or segments that coincided with binding specificity.

Among sequences conserved between FimA proteins are the N-terminal signal peptide, pilin motif (WXYDVXVYPKN), proline-rich segment (PPXPXXPPXXPXNPP), E box (LVETKAPXGX) and LPXTG sorting motif (LPLTG), two additional proline-containing segments, as well as four other segments with high sequence identity (Fig. 3A). The pilin, E-box and LPXTG motifs displayed 80 to 100% sequence identity for FimA proteins present in *A.odontolyticus *and *A. naeslundii *genospecies 1 and 2 (Fig. 3B). Multiple FimA segments showed a low sequence identity between the species.

Apart from the sequences unique to and conserved within each species, we could not identify or link any distinct motif, sequence or substitution to the different binding or hem-agglutination types between species or strains. However, the genospecies 1 strain P-5-N and genospecies 2 strain LY7 with somewhat lower intra-species sequence identities, 93.8% and 88.6%, showed unique hemagglutination or coaggregation patterns, respectively (Table 3).

### Structural comparison of FimA and FimP proteins from A. naeslundii, A. odontolyticus and A. viscosus

The FimP (from *A. naeslundii *genospecies 2, n = 2, and *A. viscosus*, n = 1) and FimA protein families contained conserved N-terminal signal peptides and C-terminal membrane spanning segments, but with low sequence identity to each other (~24 %) (Fig 3A). High identity sequences in both FimA and FimP are the proline-containing domains 2, 3, 4 (pilin motif), 6 (E box) and 7 (LPXTG). In contrast, the proline-containing regions 1 and 5 are conserved domains in FimP but show only conserved proline residues in FimA.

### FimA from A. naeslundii genospecies 1 and 2 and A. odontolyticus display different antigenic properties

**Figure 4:**
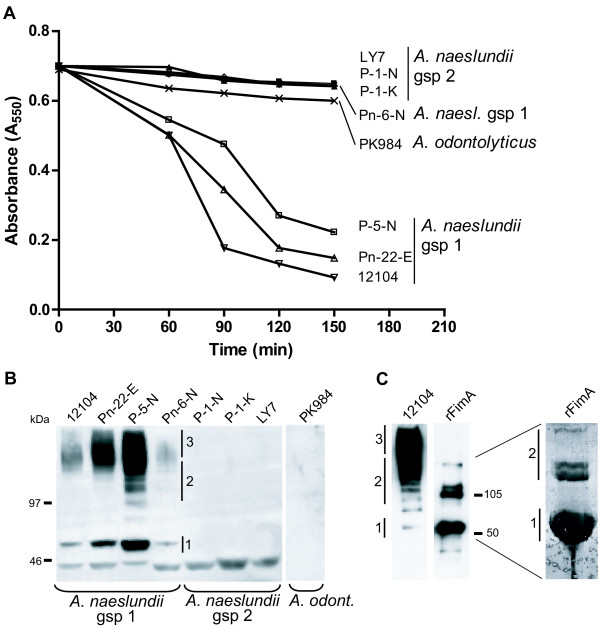
**Antigenic properties and oligomerization of native and recombinant FimA**. **A) **Whole cell agglutination by type-2 fimbriae specific antisera (derived from genospecies 1 ATCC 12104) of *A. naeslundii *genospecies 1 (strains 12104, Pn-22-E, P-5-N) but neither of genospecies 2 (strains LY7, P-1-N, P-1-K) nor of *A. odontolyticus *(strain PK984). Experiments were repeated three times with similar results. **B) **Western blots of whole cell proteins from strains of *A. naeslundii *genospecies 1 and 2 and *A. odontolyticus *PK984 with type-2 fimbriae specific antisera. FimA monomers (no. 1), oligomers (no. 2) and polymers (no. 3) are marked. **C**) Western blots of whole cell proteins from *A. naeslundii *strain 12104 and recombinant FimA from strain 12104, and Coomassie blue-stained gel electrophoresis pattern (to the right) of the mono- to oligomer region of recombinant FimA. Molecular mass markers are indicated.

Strains of *A. naeslundii *genospecies 1 and 2 and *A. odontolyticus *were tested for reactivity with antisera R70-3 specific to FimA from type-2 fimbriae of genospecies 1 strain 12104 using whole cell agglutination and Western blot analyses with whole cell proteins (Fig. 4). While all strains of *A. naeslundii *genospecies 1, except for strain Pn-6-N, were agglutinated by the antisera, neither strains of genospecies 2 nor *A. odontolyticus *PK984 were agglutinated (Fig. 4A). Thus, the native FimA protein variants possess different antigenic properties and potentially different overall folding patterns.

Moreover, only genospecies 1 strains possessed positive FimA protein bands in Western blot analyses with type 2 fimbriae specific antisera(Fig. 4). Besides the FimA monomer (no. 1) and polymers (no. 3) detected in all genospecies 1 strains, di- to oligomers of FimA (no.2) were also detactable, suggesting either oligomerization of FimA subunits or the presence of polymeric fragments of covalently tathered FimA subunits.

### Oligomerization of recombinant FimA protein

To further explore the possible oligomerization of FimA in solution, we expressed and purified the FimA protein from *A. naeslundii *strain 12104 as a recombinant protein and analysed its ability to oligomerize by gel electrophoresis and Western blot analyses using FimA specific antisera (Fig 4C). Gels of the recombinant FimA protein revealed monomers (no. 1) and oligomers (no. 2) of FimA, as confirmed by Western blotting, verifying the possibility of FimA di- to oligomerization in solution but dependence on whole cells for fimbriae polymerization.

## Discussion

The present study shows three unique subgroups of FimA proteins present in *A. naeslundii *genospecies 1 and 2 and *A. odontolyticus *with different glycoconjugate receptors. It therefore supports our hypothesis that commensal micro organisms, like the genus *Actinomyces*, exhibit complex and divergent mosaics of adhesin molecules related to species or subpopulations with different tropism and ecological niches. Notably, *A. naeslundii *genospecies 1 and 2 and *A. odontolyticus *are members of coaggregation groups A/F, B/C/D and E, respectively, and differ in a variety of type-1, type-2 and other adhesive properties [14]. The FimA protein, which contains both sequences unique to and conserved between the species, may have evolved to match specific niches in supra- or subgingival plaque or in buccal or tongue epithelial tissues. The novel FimA protein from *A. odontolyticus *strain PK984, a reference strain for *Actinomyces-Streptococcus *coaggregations typical of subgingival plaque, may mediate coaggregations or adhesive interactions typical of subgingival plaque. Strains of *A. odontolyticus *from the tongue display FimA and FimP hybridization patterns somewhat different from that observed for strain PK984 [14].

All three FimA protein variants contained a pilin motif for polymerization, E box for associated proteins and LPXTG motif for cell surface sorting and anchoring. The pilin, E box and LPXTG motifs were highly conserved among the three species, and present among the proline-containing domains suggested by Yeung and Cisar to account for folding or intermolecular interactions of FimA and FimP proteins [25]. Interestingly, the pilin, E box and LPXTG motifs are present in fimbriae proteins from *C. diphtheriae*, which have been used to express type-2 fimbriae from *A. naeslundii*, and in various other Gram-positive bacteria expressing pili-like structures [29,30]. This suggests that Gram-positive bacteria may have evolved related proteins and pathways for pili formation and function [31]. Moreover, the serologically different properties of the FimA proteins present in genospecies 1 and 2 and *A. odontolyticus *could imply that the overall folding of the FimA protein could form different functionalities or binding specificities. Actually, the variant FimA proteins did not cross-react with antisera despite many conserved motifs, which consequently may be hidden within the subunit or by intermolecular interactions. Apart from the sequences unique to each species, we could not link any FimA motifs or substitutions to the species-specific binding specificities or variant HA patterns of each genospecies. However, receptor-binding FimA subunit domains or tip-localised adhesins other than the FimA subunit remains to be identified. In this respect the unique presence of a proline-rich segment in FimA but not in FimP proteins is interesting, but of yet unknown biological significance.

Another interesting finding of the present study is the possible sortase-independent oligomerization of FimA in solution. Western blot analyses revealed FimA oligomers in whole cell protein extracts and, more importantly, mono to oligomers of FimA in purified recombinant FimA preparations. Sortase, which is absent in Gram-negative *E. coli *used for expression of recombinant FimA protein, catalyses the covalent tethering of FimA subunits through pilin and LPXTG motifs when expressed in Gram-positive *C. diphtheriae *[29]. It remains, however, to be determined whether the pilin motif and/or other conserved subunit motifs are involved in this sortase-independent ability of FimA to oligomerize in solution. Finally, it is reasonable to assume that the spontaneous ability of FimA to di- to oligomerize acts in conjunction with sortase in the whole cell-dependent process of fimbriae assembly and polymerization.

The unique and conserved nature of FimA for each *Actinomyces *species or subspecies reinforces the important role of FimA in selection of ecological niches. Moreover, the highly conserved nature of FimA within a subspecies could also indicate an immunological tolerance to this protein on *Actinomyces *species that early colonize the oral cavity of infants [3]. The conserved nature of FimA is different to the antigenic variation found in P-fimbriae-associated PapG adhesins on uropathogenic *Escherichia coli *[32]. Based on its unique and conserved nature, we have previously designed DNA probes from particular FimA segments to distinguish between clinical isolates of genospecies 1 and 2. We assume that corresponding FimA segments unique to *A. odontolyticus *could be used in a similar way to generate probes specific to particular receptor-binding subtypes of this organism.

While different Galβ specificities of genospecies 1 and 2 target the two species to glycolipid or glycoprotein receptors [20], respectively, *A. odontolyticus *PK984 recognizes sialic acid residues on glycoproteins. Both glycolipids and glycoproteins are capable of mediating adhesion and intra-cellular signalling by epithelial cells. The host responses mediated by a glycolipid or a glycoprotein receptor may be different, and hypothetically relate to the commensal or pathogenic potential of *Actinomyces *subtypes. Moreover, the Galβ and sialic acid binding specificities may target *A. naeslundii *and *A. odontolyticus *to different bacterial partners, in particular since they belong to different coaggregation groups. In this respect, it is notable that sialidase, which modifies the hemagglutination properties of *A. naeslundii *and *A. odontolyticus *in the opposite way, is produced by *Actinomyces *and other plaque bacteria [33]. However, whether *A. naeslundii *or related coaggregation communities use sialidase to compete with *A. odontolyticus in vivo *is unknown. Finally, *A. naeslundii *and *A. odontolyticus *are interesting model bacteria for studying the role of fimbriae proteins and their receptors specificities in microbial colonisation and host-microbe interactions.

## Conclusion

This report shows three unique subgroups of FimA proteins in *A. naeslundii *genospecies 1 and 2 and *A. odontolyticus*, and that each subgroup coincides with a unique carbohydrate binding specificity. All FimA proteins contained a pilin motif for polymerization, E box, and LPXTG motif for cell surface sorting and anchoring. Finally, a sortase independent oligomerization of FimA subunit proteins in solution was indicated.

## Methods

### Bacterial strains and culturing conditions

The *A. naeslundii *genospecies 1 strains Pn-22-E (CCUG 34193), Pn-6-N (CCUG 33519), P-5-N (CCUG 33914), ATCC 12104 (CCUG 32832), and *A. naeslundii *genospecies 2 strains P-1-N (CCUG 33910), P-1-K (CCUG 32838), LY7 (CCUG 33934) and T14V used were generally from the Culture Collection, University of Göteborg (CCUG), Sweden. *A.odontolyticus *PK984 was provided by Dr. Kolenbrander, National institute of health/NIDCR, Bethesda, USA. The strains were cultured on Columbia-II-agar base plates (Becton Dickinson and Co., Cockeysville, MD), supplemented with 3 % human red blood cells, at 37°C in candle jar.

### Cloning and sequencing of fimA genes

Chromosomal DNA was isolated from bacteria and purified as described [14]. Gene segments were amplified by PCR, using standard protocols, by use of primers both inside and outside the *fimA *open reading frame (primer sequences are available upon request). All *fimA *PCR fragments were cloned into pGEM-T vectors using T4 DNA ligase (Promega Corp., Madison, WI), except for *fimA *from *A. odontolyticus *for which fragments were cloned into pCR 2.1-TOPO vectors using Invitrogen TOPO TA Cloning kit (Invitrogen, Carlsbad, CA).

Plasmid DNA was isolated using the QIAprep^® ^Spin Miniprep Kit (Qiagen GmbH, Hilden, Germany) and the size of DNA inserts were subsequently confirmed by *SalI*I and *Nco*I or only *EcoR1 *(Invitrogen) cleavage. Sequencing was done using the Thermo Sequenase radiolabeled terminator cycle sequencing kit (Amersham Life Science, Cleveland, OH), and were performed according to the manufacturer's instruction. The DNA fragments were sequenced in both directions.

### Sequence and motif analysis

Complete open reading frames of *fim*A nucleotide sequences were analyzed using the Wisconsin Package (version 9.0) from the Genetics Computer Group (GCG, University of Wisconsin, Madison, WI), except for *fimA *from *A. odontolyticus *which was analysed with The Molecular Toolkit [34].

Sequence alignments and dendrogram was made using Clustal W (1.83) multiple sequence alignment [35].

Signal peptide, LPXTG motif and proline-rich region were analyzed using bioinformatics tools [36-38].

### Nucleotide sequence accession numbers

The GenBank accession numbers for genes presented in this paper are: for *fim*A: strain Pn-22-E [GenBank: DQ425098], strain Pn-6-N [GenBank: DQ425100], strain P-5-N [GenBank: DQ425097], strain P-1-N [GenBank: DQ425102], strain P-1-K [GenBank: DQ425099], strain LY7 [GenBank: DQ425101], strain ATCC 12104 [GenBank: M 21976], strain T14V [GenBank: AF019629], and strain PK984 [GenBank: DQ425103]; for *fimP*; strain P-1-K [GenBank: AF107019], strain LY7 [GenBank: AF10720], *A. viscosus *19246 [GenBank: M 21976]; for *SpaH*: *C. diphteriae *NCTC 13129 [GenBank: NP_940533].

### Agglutination of Actinomyces by antisera

Agglutination of *Actinomyces *cells by rabbit antiserum R70-3 specific for type-2 fimbriae of *A. naeslundii *strain ATCC 12104 and pre-immunization serum R70-0 was a gift from Dr. Cisar, National institute of health/NIDCR, Bethesda, USA. Antisera (1 μml) was added to *Actinomyces *cells (1 ml, OD_550 _= 0.7) suspended in coaggregation buffer (150 mM NaCl, 0.1 mM CaCl_2_, 0.1 mM MgCl_2_, 1.0 mM Tris pH 8.0 and 0.02 % sodium azide). After incubation for one hour at room temperature on slow rotation, agglutination was measured by recording the optical density at 550 nm for 90 min, giving a total agglutination time of 150 min.

### Cloning, expression and purification of recombinant FimA

The fimA gene was PCR-amplified from genomic DNA from strain 12104 and cloned into the expression vector pETM11 (EMBL, Hamburg, Germany). The resulting construct encodes a protein (recombinant FimA or rFimA) with an N-terminal hexa-histidine tag, an 18 residue long linker and the FimA protein excluding the N-terminal signal sequence and the C-terminal transmembrane helix. *E. coli *BL21 (DE3) (Novagen, Madison, WI) was transformed with the pETM11-rFimA construct and grown at 37°C to optical density (OD_600_) of 0,5. Protein expression was induced with 0.5 mM isopropyl β-D-thiogalactoside for four hours at 30°C. Cultures were harvested and the cells lysed by sonication. rFimA was purified by Ni-agarose chromatography (Quiagen) and elution with imidazole. The protein was further purified by a size exclusion on a Superdex 26/60 column (Amersham, Uppsala, Sweden). The rFimA fractions were analyzed by SDS-PAGE and by Western blot.

### Western blot of whole cell extracts and recombinant FimA

A suspension (300 μml) of bacterial cells (3 × 10^9 ^cells) was sonicated 4 × 15 seconds using a Branson sonicator (Branson ultrasonics corporation, Danbury, CT). Proteins were precipitated with acetone for 1 hour at -20°C, centrifuged and dissolved in 50 μml sample buffer (62.5 mM Tris, 10.1% glycerol, 2.0 % SDS, 5 mM dithiothreitol and 0.01% pyronin) by boiling for 10 min. After centrifugation, the supernatant was electrophoretically separated on a 4 to 20 % or 4 to 15 % (whole cell extracts and recombinant FimA, respectively) polyacrylamid gel using Tris-glycine buffer (25 mM Tris, 192 mM glycine, and 0.1 % SDS, pH 8.3) at 15 mA. The separated proteins were blotted onto membranes (Immobilon PVDF membrane, Millipore Corp., Bedford, MA) using a transblotter. The membranes were blocked with 5 % non-fat dried milk overnight and incubated with antisera R70-3 (diluted 1:20,000) overnight followed by five washes in 10 mM phosphate buffered saline (PBS), pH 6.8, 0.5 % Tween. Peroxidase conjugated goat anti-rabbit IgG was used as secondary antibody (Dakopatts a/s, Glostrup, Denmark) and a chemiluminescent substrate (Supersignal Substrate; Pierce, Rockford, Il) was used to visualize immobilized antibodies.

### Hemagglutination

Equal volumes (10 μml of each) of suspensions of bacterial cells (3 × 10^9 ^cells/ml PBS or reciprocal dilutions) and human, goat or chicken erythrocytes (4 % erythrocyte suspension in PBS) were mixed and agitated gently for 1 min on a glas slide. Erythrocytes were depleted of sialic acid by incubation with 1 unit/ml *Clostridium perfringens *neuraminidase (Sigma Chemical Co, St Louis, MO) for 30 min. at 37°C. Agglutination was scored visually; 0 = no agglutination, 1 = weak agglutination, 2 = moderate agglutination, 3 = strong agglutination, and 4 = very strong agglutination.

## Authors' contributions

MD, KH and UÖ performed laboratory analyses, analysed data and participated in writing the manuscript. AB sequenced and analysed the *fimA *gene from PK984 and KP expressed the rFimA protein. IJ and NS designed and planned the project, as well as were responsible for writing the manuscript, at an overall level.

## Figures and Tables

**Table 1:** Type-2 fimbriae subtypes on *A. naeslundii *genospecies 1 and 2 and *A. odontolyticus *with different binding properties.

	*A. naeslundii*		*A. odontolyticus*
		
	gsp 1	gsp 2	
			
	12104	LY7	PK984
Fimbriae type^a^	2:1	2:2	2:3
			
Sugar specificity^b^			
Galβ	+ (GL)	+ (GP)	-
Sialic acid	-	-	+ (GP)
			
Hemagglutination^c^			
Human	1	0	4
Human + sialidase	3	4	1

^a^Subtypes of type-2 fimbriae, types-2:1 to 2:3, among Actinomyces species based on different hybridization patterns of strains with fimA-derived DNA probes and binding patterns to carbohydrates, red blood cells and bacterial partners [14].

^b ^Saccharide specificity and receptor ligand (GL= glycolipid, GP = glycoprotein) for each fimbriae subtype or reference strain [20, 28].

^c ^Hemagglutination (HA) patterns for each fimbriae subtype or reference strain. Score 0 marks no HA and score 4 strong HA. Similar HA results were obtained with goat and chicken erythrocytes (data not shown).

**Table 2:** Inter- and intra-species sequence identity of FimA subunit proteins^a^

	*A. naeslundii*		*A. odontolyticus*
		
Strains^b^	Genospecies 1 12104 FimA 2:1	Genospecies 2 T14V FimA 2:2	PK984 FimA 2:3
*A. naeslundii *gsp 1 (n = 4)	93.8–97.6	62.8–64.1	63.7–65.2
*A. naeslundii *gsp 2 (n = 4)	63.8–64.4	88.6–99.6	64.9–66.4

^a ^Sequence identity (%) of strains as compared to strains 12104, T14V and PK984. FimA 2:1 to 2:3 denotes FimA proteins associated with the type-2 fimbrial subtypes 2:1 to 2:3 with different binding patterns to carbohydrates, red blood cells and bacterial partners [14, 20, 28].

^b ^Genospecies 1 strains were 12104, Pn-22-E, Pn-6-N and P-5-N, and genospecies 2 strains were T14V, P-1-N, P-1-K and LY7.

**Table 3:** Sequence identity of FimA proteins within *A. naeslundii *genospecies 1 and 2 and associated binding properties.

		Sequence identity (%)^a^	HA pattern^b^				HA type^c^	COG group^d^
					
Strains			Human(+Na)	Porcine	Human	Horse		
*A. naeslundii *gsp 1								
	12104	100	1/8	1/4	1/2	1/8	a	B
	Pn-22-E	97.6	1/32	1/16	1/4	1/8	a	
	Pn-6-N	97.2	1/32	1/16	1/2	1/32	a	
	P-5-N	93.8	1/16	1/4	0	0	b	
								
*A. naeslundii *gsp 2								
	T14V	100	1/16	0	0	0	a	A
	P-1-N	99.3	1/16	0	0	0	a	
	P-1-K	99.6	1/32	1/16	0	1/4	b	
	LY7	88.6	1/32	1/16	0	1/4	b	F

^a^Amino acid sequence identity (%) within each genospecies as compared to strains 12104 or T14V, respectively.

^b^Hemagglutination (HA) of neuraminidase treated human and untreated porcine, human and horse red blood cells by reciprocal dilutions of bacterial cell suspensions. The lowest dilution with visible HA is given. 0 marks no agglutination even at the highest dilution.

^c^a and b denotes the two hemagglutination patterns observed in each genospecies.

^d^Coaggregation properties as defined by coaggregation groups (COG) A to F [27]
